# RNA splicing regulated by RBFOX1 is essential for cardiac function in zebrafish

**DOI:** 10.1242/jcs.166850

**Published:** 2015-08-15

**Authors:** Karen S. Frese, Benjamin Meder, Andreas Keller, Steffen Just, Jan Haas, Britta Vogel, Simon Fischer, Christina Backes, Mark Matzas, Doreen Köhler, Vladimir Benes, Hugo A. Katus, Wolfgang Rottbauer

**Affiliations:** 1 Department of Medicine III, University of Heidelberg, D-69120 Heidelberg, Germany; 2 German Centre for Cardiovascular Research (DZHK), partner site Heidelberg/Mannheim, D-69120 Heidelberg, Germany; 3 Chair for Clinical Bioinformatics, Saarland University, D-66123 Saarbrücken, Germany; 4 Department of Human Genetics, Saarland University, D-66123 Saarbrücken, Germany; 5 Department of Medicine II, University of Ulm, D-89081 Ulm, Germany; 6 Comprehensive Biomarker Center, D-69120 Heidelberg, Germany; 7 EMBL, European Molecular Biology Laboratory, Genomics Core Facility, D-69117 Heidelberg, Germany

**Keywords:** Dilated cardiomyopathy, Genetics, Zebrafish, Deep sequencing, Splicing

## Abstract

Alternative splicing is one of the major mechanisms through which the proteomic and functional diversity of eukaryotes is achieved. However, the complex nature of the splicing machinery, its associated splicing regulators and the functional implications of alternatively spliced transcripts are only poorly understood. Here, we investigated the functional role of the splicing regulator *rbfox1 in vivo* using the zebrafish as a model system. We found that loss of *rbfox1* led to progressive cardiac contractile dysfunction and heart failure. By using deep-transcriptome sequencing and quantitative real-time PCR, we show that depletion of *rbfox1* in zebrafish results in an altered isoform expression of several crucial target genes, such as *actn3a* and *hug.* This study underlines that tightly regulated splicing is necessary for unconstrained cardiac function and renders the splicing regulator *rbfox1* an interesting target for investigation in human heart failure and cardiomyopathy.

## Abstract (precis)

**Summary:** The zebrafish splicing regulator *rbfox1* is necessary for tightly regulated splicing and unconstrained cardiac function, making it an interesting candidate for targeting during human cardiomyopathy.

## INTRODUCTION

Alternative transcript initiation, alternative splicing and alternative polyadenylation of pre-mRNAs are considered to be key mechanisms for generating the huge proteomic diversity in eukaryotic species (Griffith et al., 2010). At least >95% of human genes are estimated to be alternatively spliced in a tissue- or cell-type-dependent manner (Johnson et al., 2003; Pan et al., 2008; Wang et al., 2008), allowing the cell to act in a certain environment or adopt distinct tissue-specific functions. Regulation of alternative splicing builds upon a complex splicing machinery that joins both constitutive and regulated exons within nuclear pre-mRNA molecules. Removal of introns from pre-mRNAs is carried out by a large macromolecular machine that is known as the spliceosome, which includes five small nuclear ribonucleoproteins (snRNPs; U1,U2, U4, U5 and U6) and several hundred associated regulator proteins (Wahl et al., 2009). Some of the best-characterized splicing regulators include the serine-arginine (SR)-rich family, heterogeneous nuclear ribonucleoproteins (hnRNPs) proteins, and the Nova1 and Nova2, and the PTB and nPTB (also known as PTBP1 and PTBP2) families (David and Manley, 2008; Gabut et al., 2008; Li et al., 2007; Matlin et al., 2005). The diversity in splicing is further increased by the location and nucleotide sequence of pre-mRNA enhancer and silencer motifs that either promote or inhibit splicing by the different regulators. Adding to this complexity, regulating motifs are very common throughout the genome, but are not necessarily functional (Barash et al., 2010; Black, 2003; Fairbrother et al., 2002; Wang et al., 2004; Zhang and Chasin, 2004).

Defective splicing of transcripts has been related to disturbed embryonic development, cellular de-differentiation and the pathogenesis of several diseases (Ars et al., 2000; Creemers et al., 2006; Davis et al., 2002; Disset et al., 2006; Kalsotra et al., 2008; Kong et al., 2010; Pasumarthi and Field, 2002; Tang et al., 2012, 2009). Although such alterations in the splicing machinery have mainly been related to cancer or mental disorders (Cork et al., 2008; French et al., 2007; Klinck et al., 2008), they also play a role in the development and progression of heart diseases, such as heart failure and cardiac hypertrophy. For example, aberrant splicing of cardiac troponins is linked to the progression of cardiomyopathies and heart failure (Biesiadecki et al., 2002; Biesiadecki and Jin, 2002; Philips et al., 1998). Expression of an alternatively spliced variant of the sarcomere gene titin (*TTN*) has been associated with progression of chronically ischemic heart failure and differentially spliced *MYH7* and *FLNC* genes are found at high copy numbers in failing or hypertrophied hearts (Davis et al., 2002; Kong et al., 2010; Neagoe et al., 2002). Recently, Guo et al. have also shown that the splice factor RBM20 is strongly expressed in cardiac tissue, and transcriptome analysis in humans and rats has revealed RBM20-mediated regulation of alternative splicing events in a set of relevant cardiac genes (Guo et al., 2012), demonstrating the importance of stoichiometrically correct isoform expression. Reduced activity of RBM20 results in an altered isoform expression of cardiac proteins crucial for proper heart structure and function (Carboni et al., 2010).

Recent studies have shown that members of the so-called RBFOX family, such as *rbfox1-like* and *rbfox2*, also play an important role in mRNA splicing (Gallagher et al., 2011). Using the zebrafish (*Danio rerio*) as a model system to study vertebrate development, biological processes and disease mechanisms, we investigated the functional role of the splice factor *rbfox1* in the vertebrate heart. By RNA sequencing and real-time PCR validation, we detected and delineated a functional role for new *rbfox1* splicing targets *in vivo*.

## RESULTS

### The RNA-splicing regulator *rbfox1* is highly expressed in the zebrafish heart

The *rbfox1* gene encodes a 373-amino-acid protein containing an RNA-binding domain (RBD) motif that is highly conserved among RNA-binding proteins. It is one of four *rbfox* paralogs in zebrafish, which also include *rbfox1l* (also known as *fox-1*, Ch16; Entrez Gene ID 406569), *rbfox2* (also known as *rbm9*, Chr6; Entrez Gene ID 407613) and *rbfox3* (Chr3; Entrez Gene ID LOC559412). Each member of the RBFOX protein family specifically binds to (U)GCAUG elements and regulates alternative splicing positively or negatively in a position-dependent manner (Jin et al., 2003; Nakahata and Kawamoto, 2005; Underwood et al., 2005; Zhang et al., 2008). In detail, they promote exon inclusion when binding to the intron downstream from an alternative cassette exon, and exon skipping when binding to the upstream intron (Auweter et al., 2006; Tang et al., 2009).

**Fig. 1.**
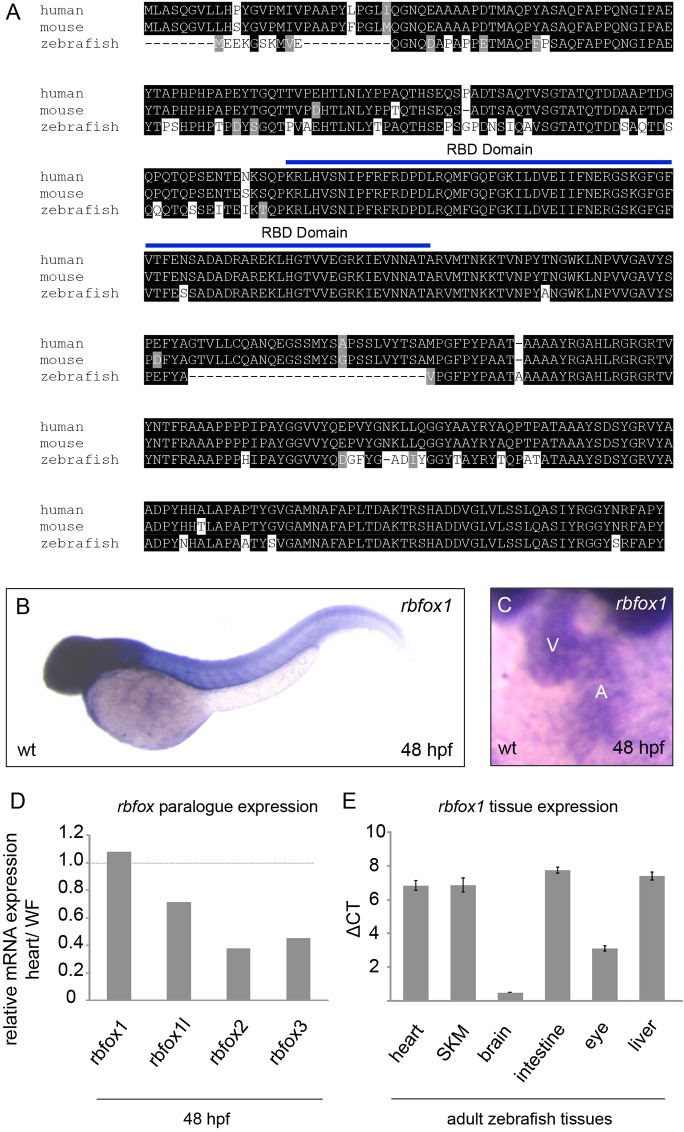
**Expression analysis of *rbfox1* in zebrafish.** (A) Amino acid sequence alignments of human, mouse and zebrafish *rbfox1* demonstrating the high cross-species homology. Black background, amino acid identity; gray background, amino acids with similar chemical properties. (B,C) RNA antisense *in situ* hybridization against *rbfox1* demonstrates a specific mRNA expression in neuronal, heart cells and skeletal muscle at 48 hpf (V, ventricle; A, atrium). wt, wild-type. (D) Relative expression analysis of *rbfox* mRNAs shows that *rbfox1* is highly expressed in the heart in comparison to the other *rbfox* family members. WF, whole fish. (E) qRT-PCR analysis of *rbfox1* in different tissues of adult zebrafish reveals the highest expression in brain and eye tissue, and an equal expression in heart, skeletal muscle (SKM), intestine and liver tissue. Data are shown as mean±s.d. (pooled tissue of adult zebrafish *n*=8, two replicates each). ΔCt values compared to *elfa1* as a reference gene.

The protein sequence identity between the zebrafish and human ortholog is ∼84% (Fig. 1A). Characterization of *rbfox1* gene expression by whole-mount *in situ* hybridization and quantitative real-time PCR (qRT-PCR) revealed that *rbfox1* was expressed in all tissues at 48 hours post fertilization (hpf, Fig. 1B), consistent with previous studies (Kurrasch et al., 2007; Thisse, 2004; Gallagher et al., 2011). Using whole-mount *in situ* hybridization, qRT-PCR and RNA-Seq we could show that there was considerable expression of *rbfox1* in the zebrafish heart at 48 hpf (Fig. 1C–E). Interestingly, *rbfox1* transcripts were, in comparison to the other *rbfox* paralogs, *rbfox1l*, *rbfox2* and *rbfox3*, more abundant in heart tissue at 48 hpf compared to whole-fish tissue expression (Fig. 1D). qRT-PCR analysis further showed that *rbfox1* was expressed in the adult in all investigated tissues (Fig. 1E).

### Loss of *zrbfox1* leads to impaired cardiac function

**Fig. 2.**
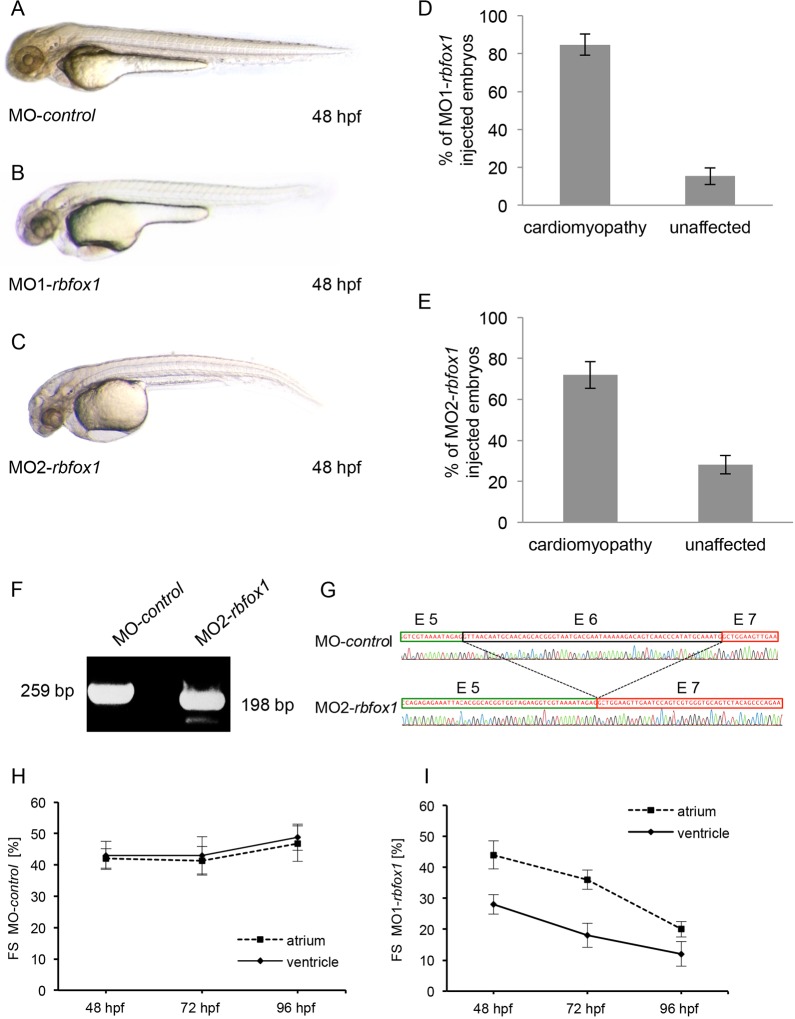
**Knockdown of *rbfox1* leads to cardiomyopathy and heart failure.** (A–C) Lateral view of MO-*control*- and MO1- and MO2-*rbfox1*-injected embryos. After injection of 3 ng MO1-*rbfox1*, 85% (D) and MO2-*rbfox1*, 72% (E) of morphants embryos develop cardiomyopathy and heart failure. Results are mean±s.d. (*n*=3). (F) cDNA analysis of *rbfox1* morphants after injection of MO2-*rbfox1* shows skipping of exon 6 (198-bp product) compared to a product including exon 6 (259 bp product) in control-treated embryos. Sanger sequencing reveals that exon 6 is completely excluded, predictably leading to a frame shift of the coding sequence and premature stop in exon 7. (H,I) Fractional shortening (FS) of the ventricular chamber of MO-*control*- and MO1-*rbfox1-*injected embryos measured at the indicated developmental stages. Fractional shortening is significantly reduced in *rbfox1* morphants after 48 hpf and further declines at 96 hpf. Results are mean±s.d. (*n*=10).

A cardiac role for *rbfox1* had not been demonstrated, hence our aim was to investigate the functional role of this new splicing regulator *in vivo* by using the zebrafish as a model organism. To do so, we first inactivated zebrafish *rbfox1* by injecting morpholino (MO) antisense oligonucleotides directed against either the translational start-site (MO1) or splice donor site of intron-5–exon-6 (MO2) into one-cell-stage zebrafish embryos. To monitor the effect of the splice morpholino, we performed cDNA splice-site analysis and found that blockage of the splice donor site of exon 6 of *rbfox1* resulted in a complete exclusion of exon 6 (Fig. 2F). Absence of exon 6 leads to a frameshift of the coding sequence and to a predicted truncated protein due to a premature stop within exon 7 (Fig. 2G). As a result of the *rbfox1* knockdown, 85% of the zebrafish embryos injected with MO1 (*n*=159; Fig. 2D) and 72% of those injected with MO2 (*n*=100, Fig. 2E) developed progressive heart failure due to continually decreasing ventricular contractility as measured by fractional shortening (Fig. 2B,C; supplementary material Movie 2). In detail, the ventricular contractility of morphants decreased progressively from 28±3.2% at 48 hpf to 18±3.9% (mean±s.d.) at 72 hpf (Fig. 2I). By ∼96 hpf, both chambers collapsed and became almost silent. Consequently, blood flow completely arrested. Embryos injected with a control morpholino (MO-*control*) did not show any morphological or functional abnormalities (Fig. 2A,H; supplementary material Movie 1). In addition to the observed cardiac defect, we only noticed the development of a slightly brain edema.

**Fig. 3.**
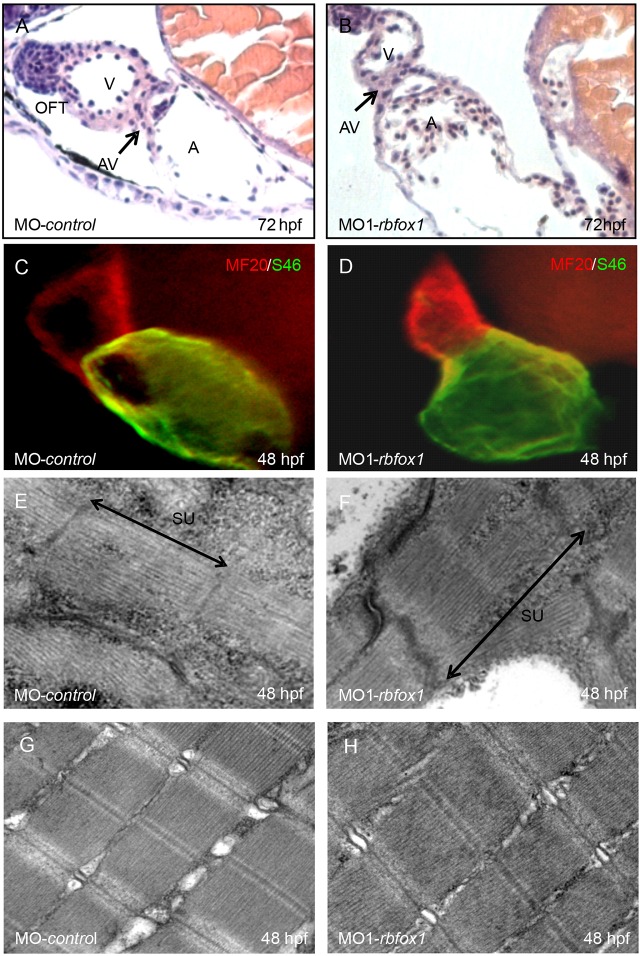
***rbfox1* deficency does not influence heart morphology.** (A,B) H&E-stained sagittal histological sections of MO-control and MO1-*rbfox1* morphant hearts at 72 hpf. Morphants display normal heart morphology with distinct endocardial and myocardial cell layers in the atrium (A) and ventricle (V), and a clear differentiation and demarcation of the atrium and ventricle by the atrio-ventricular ring (AV). OFT, outflow tract. (C,D) Atrial- and ventricle-specific myosin heavy chains are expressed normally, also suggesting that there is normal molecular chamber specification [green, antibody against atrial-specific myosin (S46); red, antibody against ventricular and atrial myosin (MF20)]. (E–H) Ultrastructural analysis of heart and skeletal muscle cells of MO-*control*- and MO1-*rbfox1*-injected embryos at 48 hpf showing organized sarcomere units (SU) with thin and thick myofilaments in well-aligned bundles and discernible AI-, M- and Z-bands.

To evaluate further the causative mechanism responsible for the severe heart failure phenotype observed in *rbfox1* morphants, we next analyzed their cardiac morphology and ultrastructure. Histological analysis showed that the cardiac chambers of the *rbfox1*-morphant hearts were well defined and separated by the atrio-ventricular ring (Fig. 3A,B), and that the endocardial and myocardial layers of both chambers had developed properly with a multilayered ventricular myocardium. Occasionally, we also observed morphants displaying atrio-ventricular-canal (avc) malformations, potentially due to the pronounced dilation of the atrium. On the molecular level, atrial and ventricular cardiomyocytes of *rbfox1*-morphants expressed myosin heavy chains in the typical heart-chamber-specific pattern, suggesting that there was normal molecular chamber specification (Fig. 3D). Furthermore, transmission electron microscopy (TEM) revealed apparently normally developed cardiac and skeletal muscle sarcomeres (Fig. 3E–H) in *rbfox1* morphant embryos. Specifically, we found well-organized myofilaments, interconnected by Z-disks and aligned in discernible AI- and M-bands.

In summary, we find no structural or developmental abnormalities that sufficiently could explain the development of heart failure in *rbfox1* morphants. Hence, *rbfox1* deficiency leads to a functional defect rather than structural abnormalities. Therefore, we hypothesized that *rbfox1* proteins regulate an alternative splicing program that is essential for normal heart function during zebrafish development and that a reduced activity of *rbfox1* might result in an altered isoform expression of proteins essential for maintenance of cardiac function.

### Characterization of the cardiac transcriptome in the zebrafish by RNA deep-sequencing

To gain insights into heart-specific splicing, we first analyzed the global exon expression of genes in zebrafish hearts compared to their expression in the whole fish. For this purpose, we generated sequencing libraries from pooled control embryos at 48 hpf. In detail, we isolated RNA from *n*=70 zebrafish embryos and *n*=400 isolated purified embryonic zebrafish hearts. Next, we applied SOLiD deep sequencing and mapped the resulting 50-bp reads to the zebrafish reference genome. The normalized reads were quantified, and a mean expression was calculated for each gene. We found several transcripts that were highly abundant in the zebrafish heart but not in the whole fish and vice versa (supplementary material Fig. S1A). Supplementary material Fig. S1B,C exemplarily shows two clusters of genes that were highly expressed genes in whole fish (blue) and/or heart tissue (red), which include genes such as *tmp4*, *nppa* and *myl7*. To integrate the information from the complex deep sequencing data at a functional level, we next performed a gene set enrichment analysis for the differentially expressed genes based on the Kyoto Encyclopedia of Genes and Genomes (KEGG) definition. We found that genes with functions in mitogen-activated protein kinase (MAPK) signaling, Ca^2+^ signaling, focal adhesion and insulin signaling tended to be more highly represented in hearts, whereas those with functions in metabolic pathways, oxidative phosphorylation, ribosome, spliceosome, proteasome were less represented (supplementary material Table S1).

To characterize the diversity of the zebrafish transcriptome, we next investigated all sequencing reads of the heart and whole body that non-ambiguously matched predicted exon–exon junctions (for details, see the Materials and Methods section). Among the detected genes in the whole fish, 86% have only one isoform, 11% two isoforms, and 4% three or more isoforms (supplementary material Fig. S1D), delineating the transcriptomic diversity caused by alternative splicing mechanisms. All identified genes that undergo alternative splicing are listed in supplementary material Table S5.

### RNA-sequencing analysis identifies a specific set of *rbfox1*-dependent splicing events in zebrafish hearts

**Fig. 4.**
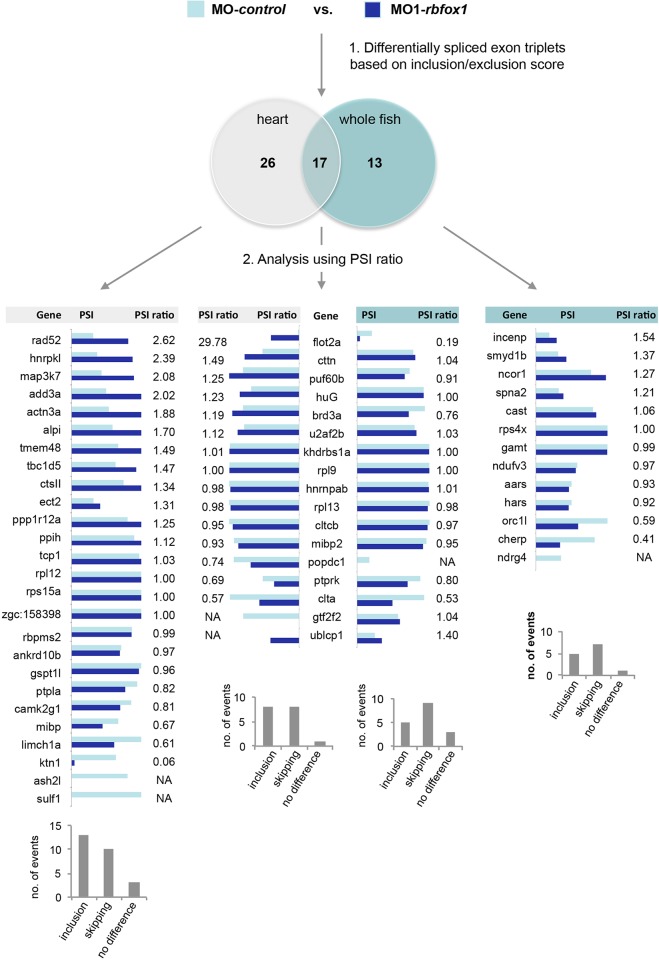
**RNA sequencing analysis of MO-*control*- and MO-*rbfox1-*injected zebrafish embryos.** Venn diagram showing differentially spliced exon triplets in heart and whole-fish tissue comparing MO-*control* and MO1-*rbfox1* morphants based on an inclusion and exclusion scoring algorithm (1). Next (2), alternative exon usage is presented as PSI values (colored bar graphs) in *control-* and *rbfox1*-depleted zebrafish. In addition, the PSI ratio is given (PSI MO1-*rbfox1*:MO-*control*). Bar graphs at the bottom detail the proportion of exon inclusion and skipping.

We next asked whether defective mRNA splicing could be responsible for the functional defects observed in *rbfox1*-deficient embryos. To do so, we evaluated the effect of reduced *rbfox1* activity on mRNA processing on a global scale. mRNA deep-sequencing of heart and whole-fish tissue from MO-*control*- and MO1-*rbfox1*-injected embryos at the 48 hpf stage was performed as described above. Owing to the predicted nature of *rbfox1*-mediated splicing, we analyzed alternative splicing changes on a single-exon usage basis. This means that splicing events lead to single exon inclusion or exclusion in the mature mRNA. The stringent analysis of transcript variants between control and *rbfox1*-depleted whole fish and heart tissue revealed 56 alternative splicing events showing differential exon usage. In detail, we found 26 differentially spliced transcripts exclusively in heart tissue, 13 in whole-fish tissue and 17 in both tissues (Fig. 4).

To determine internal exon inclusion levels, we next relied on the percentage splicing index (PSI or Ψ) approach as described previously (Wang et al., 2008), where the fraction of mRNA that contains an exon, the ‘percentage spliced in’ value, can be estimated as the ratio of the density of inclusion reads to the sum of the densities of inclusion and exclusion reads. A PSI value of 100% means that the exon is fully included and, accordingly, a value of 0% means that the exon is never included.

Fig. 4 gives the identified transcripts in heart and whole-fish tissue and the corresponding PSI values for MO-*control* and MO1-*rbfox1*. In heart tissue, we found 26 differentially spliced transcripts after *rbfox1* depletion, showing PSI ratios between 2.62 to 0.06. Among the 26 identified transcripts, 13 showed increased inclusion and ten increased exclusion, whereas three showed no difference. In whole-fish tissue, we identified 13 differentially spliced transcripts, with most of these transcripts showing exon skipping. For the 17 differentially spliced transcripts identified in both heart and in whole-fish tissue, we partially observed a discordant exon usage behavior (Fig. 4, middle panel). For instance, for *flot2a*, we observed an increased level of exon inclusion in heart tissue after *rbfox1* knockdown, whereas in whole-fish tissue we detected an almost complete exclusion of this exon.

### Validation of differential splicing in new *rbfox1* target transcripts

For the validation experiments, we assayed 22 of the 26 identified transcripts from heart tissue using qRT-PCR. Here, we also focused on alternative exon usage (inclusion or exclusion). To do so, we applied two strategies to assess the splicing pattern. First, we performed a flanking qRT-PCR, which uses primers that hybridize to the constitutive exons flanking the predicted alternative spliced exon. Second, we carried out a PCR with primers targeting the flanking constitutive exons as well as the alternative spliced exon. For normalization, constitutive exon quantification was applied. The sensitivity and the specificity of this procedure were controlled by a spike-in control. For quantification, three biological replicates were used, with *n*=400 dissected embryonic hearts each. All analyses were performed at the 48 hpf stage.

**Fig. 5.**
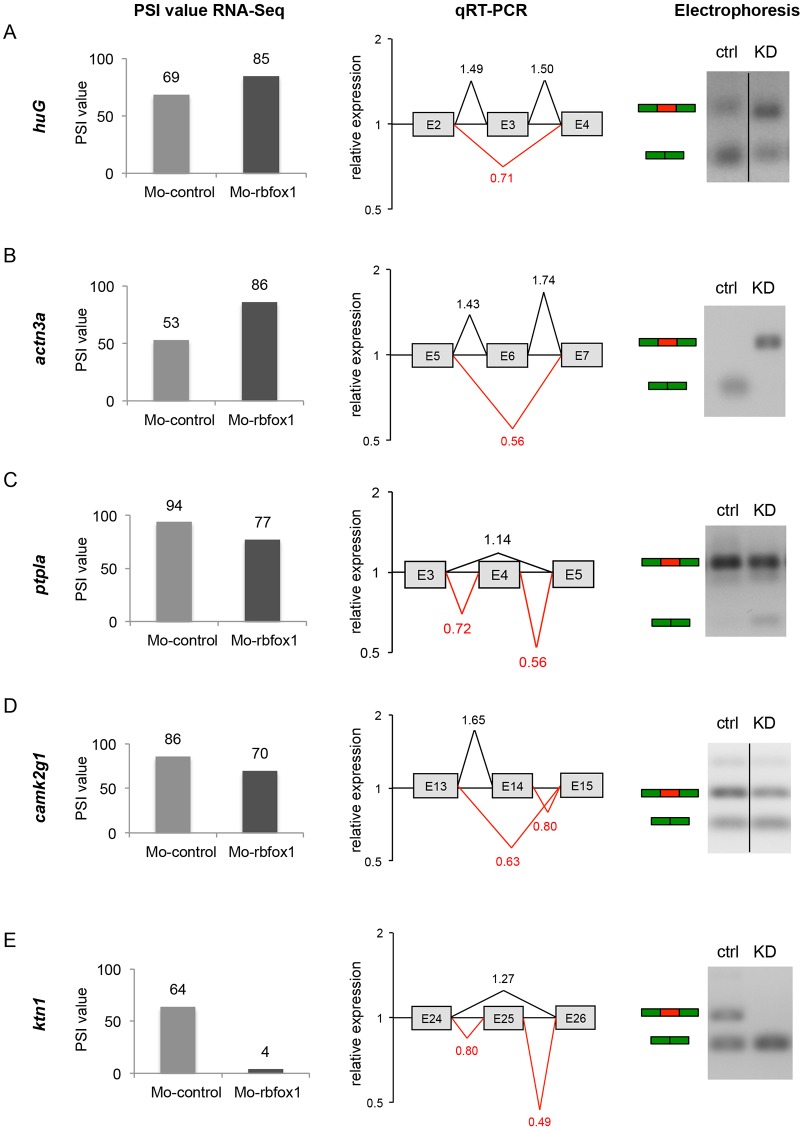
**Validation of differential splicing of *huG*, *actn3a*, *ptpla*, *camk2g1* and *ktn1*.** (A–E) Left panels show the PSI value from RNA sequencing of MO-*control* and MO1-*rbfox1* morphants. Middle panels show the relative expression of differentially spliced exons measured by qRT-PCR. Relative expression analysis in heart tissue indicates downregulation (red lines) or upregulation (black lines) of exon–exon junctions after depletion of *rbfox1*. Right panels show gel electrophoresis showing the PCR products (ctrl, control; KD, MO1-*rbfox1* morphant). Green and red boxes indicate forms with exon inclusion or exon skipping, respectively.

From the subset of 22 cardiac transcripts, five genes with alternative exon usage events were concurrently validated by the qRT-PCR and gel electrophoresis (Fig. 5A–E). These genes were *huG*, *actn3a*, *ptpla*, *camk2g1* and *ktn1*, which were among the genes with strongest differences in the PSI-based RNA sequencing analysis. In detail, we found an increased exon inclusion in *huG* and *actn3a* in heart tissue of *rbfox1* morphants. Here, qRT-PCR analysis showed a 1.5-fold (*huG* transcript) and 1.6-fold (*actn3a* transcripts) increased inclusion of the alternative spliced exon in *rbfox1*-depleted zebrafish hearts in comparison to the control-injected hearts. For *ptpla*, *camk2g1* and *ktn1*, we detected the opposite, namely an increased exon exclusion, with kinectin (*ktn1*) showing the largest observed effect.

### Functional delineation of disturbed mRNA splicing in *rbfox1* morphants

To address the question of whether the differentially spliced transcripts identified in heart tissue after depletion of *rbfox1* could partially explain the observed phenotype, we analyzed the functional role of two candidates by a reverse genetic approach.

**Fig. 6.**
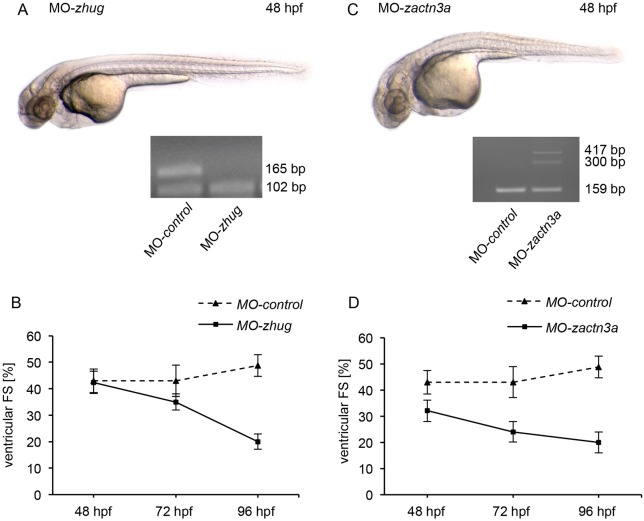
**Correctly balanced *huG* and *actn3a* isoform expression is essential for proper heart function.** (A,B) Lateral view of a MO-*huG*-injected (*MO-zhug*) zebrafish embryo at 48 hpf. Fractional shortening (FS) shows a progressive reduction of cardiac contractility. Inset, cDNA splice-site analysis of *huG* morphant. (C,D) Splice-site blockage in *actn3a* leads to mild cardiac dysfunction. *actn3a*-depleted zebrafish embryos develop heart failure with dilation of the atrium and reduced ventricular contractility beginning at the 72 h developmental stage. Inset, cDNA splice-site analysis of *actn3a* morphant. Results in B and D are mean±s.d. (*n*=10).

For this purpose, we designed specific antisense oligonucleotides targeting the alternative spliced exon of *huG* and *actn3a* to disturb their regular isoform expression. Injection of 3 ng of MO-*huG* in zebrafish embryos led to a similar but weaker cardiac phenotype to that observed after depletion of *rbfox1*. Specifically, MO-*huG*-injected embryos developed late-onset heart failure with dilation of the both chambers and pericardial edema (Fig. 6A; supplementary material Movie 3). Fractional shortening measurements showed a significantly reduced ventricular contractility, decreasing from 42±4% at 48 hpf to 20±3% (mean±s.d.) at 96 hpf (Fig. 6B). In addition to their cardiac phenotype, *huG* morphants displayed a slight swelling of the fore-, mid- and hind-brain. The conducted cDNA splice-site analysis showed that there was exclusion of exon 3 in the morphants. Nevertheless, loss of exon 3 led to a reduced cardiac function.

For *actn3a*, we found that induction of splice-site blockage at exon-6–intron-6 led to mild cardiac dysfunction. *actn3a*-depleted zebrafish embryos developed heart failure with dilation of the atrium and reduced ventricular contractility beginning at the 72 h developmental stage (fractional shortening was 35±3% at 72 hpf and 20±3% at 96 hpf) (Fig. 6C,D; supplementary material Movie 4). In addition, *actn3a*-morphants showed a reduction of blood flow and pericardial blood congestion as a consequence of the reduced cardiac function. Beside cardiac functional defects, we saw a slightly impairment of skeletal muscle function as assessed by a reduced touch response (data not shown). cDNA splice-site analysis of *actn3a* morphants revealed incorrect spliced *actn3a* transcripts, with partial inclusion of intron 6.

## DISCUSSION

RNA splicing is crucial for many biological processes and is regulated by complex mechanisms involving numerous RNA-binding proteins. To date, a large number of human diseases have been found to result from disturbed RNA splicing, most often caused by intronic or exonic mutations that disrupt splicing sites or splice-factor binding patterns. Such splicing abnormalities have been found to be particularly prevalent in neurodegenerative diseases and cancer (Cartegni et al., 2002, 2006; Cartegni and Krainer, 2002; Cooper et al., 2009; Kashima and Manley, 2003; Talbot and Davies, 2001). For instance, neurofibromatosis type I is caused by mutations in the neurofibromin 1 gene (*NF1*), and in 50% of the patients the identified mutations result in splicing alterations (Ars et al., 2000). In the heart, physiological changes during the postnatal remodeling require extensive transcriptional and posttranscriptional changes, including alternative splicing mechanisms (Kalsotra et al., 2008; Olson, 2006). This has led to speculations that splicing also contributes to maladaptive remodeling during different heart diseases. As such, mutations in the splice factor RBM20 have recently been associated with human dilated cardiomyopathy (Brauch et al., 2009; Li et al., 2010; Refaat et al., 2012). To gain further insights into the relevant mechanisms, Guo et al. used deep transcriptome sequencing to identify sets of genes with conserved splicing regulation between humans and rats, rendering animal models suitable to investigate human splicing defects (Carboni et al., 2010). Consequently, other RNA-binding proteins such as CELF-3, CELF-5, RBM24, RBM25 and LUC7L3 have been linked to the development of cardiomyopathies (Brauch et al., 2009; Gao et al., 2011; Li et al., 2010; Musunuru, 2003; Poon et al., 2012; Refaat et al., 2012). In the present study, we successfully applied a similar approach in zebrafish to dissect the role of *rbfox1* in cardiac splicing. Our results render Rbfox1, as well as its validated targets, new candidates for involvement in human cardiomyopathy and heart failure.

The transcriptome sequencing conducted in the present study indicates that 14% of all genes expressed in the heart undergo alternative splicing by single exon skipping or inclusion. This amount of alternative splicing is significantly lower than predicted in humans (Johnson et al., 2003; Wang et al., 2008). However, we only evaluated splicing events that target ‘an exon in the middle’. Hence, the actual diversity of the zebrafish transcriptome will be considerably higher. A major problem in quantifying mRNA levels by RNA sequencing is that shorter reads do not always map uniquely to a single gene and the depth of coverage considerably influences the relative quantification performance (Tarazona et al., 2011). To circumvent these limitations, we applied a two-step approach. First, we identified isoforms by deep sequencing and then quantified their expression by qRT-PCR. For the latter, Vandenbroucke et al. proposed the usage of boundary-spanning primers to quantify isoforms that strongly differ in abundance (Vandenbroucke et al., 2001). By this stepped approach, we could detect five genes with substantially changed mRNA splicing, all of which could explain the observed cardiac phenotype after depletion of *rbfox1*.

The splicing factor *rbfox1* is a member of the RBFOX-protein family, which is characterized by a common evolutionary highly conserved RNA recognition motif (RRM), flanked by less conserved N- and C-terminal domains unique for this proteins. In mammals, there are three RBFOX paralogs: RBFOX-1 (also known as ataxin2-binding protein 1), RBFOX-2 (also known as RNA-binding motif 9, RBM9) and RBFOX-3 (also known as hexaribonucleotide-binding protein 3, HRNBP3, and NeuN) (Kuroyanagi, 2009). Each member of the RBFOX protein family specifically binds to (U)GCAUG elements and regulates alternative splicing positively or negatively in a position-dependent manner (Jin et al., 2003; Nakahata and Kawamoto, 2005; Underwood et al., 2005; Zhang et al., 2008). In detail, they promote exon inclusion when binding to the intron downstream from an alternative cassette exon, and exon skipping when binding to the upstream intron (Auweter et al., 2006; Tang et al., 2009). Several human mutations have previously been mapped to this locus, with patients exhibiting severe neuro-developmental phenotypes, including mental retardation, epilepsy and autism spectrum disorder (Barnby et al., 2005; Bhalla et al., 2004; Martin et al., 2007; Sebat et al., 2007). Downregulation of *rbfox1* during cardiac hypertrophy leads to altered splicing events in mice hearts (Park et al., 2011). Here, we found that loss of *rbfox1* does not result in a completely splicing failure, but rather alters the splicing pattern of specific target genes. On a functional level, we found that *rbfox1* is necessary for cardiac contractility, potentially by regulating the correct splicing of the here identified downstream targets. However, although several targets were mis-spliced it still remains difficult to pinpoint the precise effect of each of them. Hence, we analyzed the functional role of two candidates by a reverse genetic approach. Using this approach, we showed that *actn3a* and *huG* might be downstream functional effectors and that mis-splicing caused by *rbfox*-depletion results in a cumulative phenotypic effect due to several splicing abnormalities. In case of *huG* there was, however, a difference in the splicing pattern after *rbfox1*-depletion compared to the splice-site blocking antisense probe, suggesting that a quantitatively correct isoform expression is necessary for unconstrained cardiomyocyte function. Taken together with results from previous reports, this highlights the importance of balanced mRNA splicing in the heart and represents intriguing opportunities for novel therapeutic approaches (Bauer et al., 2010; Hammond and Wood, 2011; Wood et al., 2010).

The potential impact of RNA splicing in human disease is undoubtable. It will be crucial to leverage our understanding of the biological role of alternative splicing to develop novel diagnostic and therapeutic strategies. Genetic therapies will be needed to correct RNA mis-splicing, and early studies in this area have progressed from the cell culture to promising clinical trials (Wood et al., 2010).

## MATERIALS AND METHODS

### Zebrafish strains

Care and breeding of zebrafish (*Danio rerio*) were as described previously (Meder et al., 2011). The present study was performed under institutional approvals, which conform to the Guide for the Care and Use of Laboratory Animals published by The US National Institute of Health (NIH Publication No. 85-23, revised 1996).

### Whole-mount *in situ* hybridization, histology and transmission electron microscopy

Embryos were fixed in 4% paraformaldehyde or DENT's fix solution, respectively. For histological analysis 5-µm sections were cut, dried and stained with hematoxylin and eosin (H&E) (Rottbauer et al., 2001). Whole-mount *in situ* hybridization of zebrafish embryos was conducted as previously described (Thisse and Thisse, 2008) using a digoxigenin-labeled *rbfox1* antisense probe. For TEM, embryos were fixed as described previously (Majumdar and Drummond, 1999), embedded in Epon 812 (Polysciences) and sectioned. Thin sections were cut on a Reichert Ultracut E ultramicrotome and collected onto Formar-coated slot grids. Sections were poststained with uranyl acetate and lead citrate and viewed in a Philips CM10 electron microscope at 80 keV.

### Antisense-mediated knockdown

Morpholino-modified antisense oligonucleotides (Gene Tools, Philomath, OR) used were as follows: zebrafish *rbfox1*-MO1 (ENSDARG00000014746) targeting the start site +1 to +28 (5′-ATGGAGGAAAAAGGGAGCAAGATGGTGG-3′), and *rbfox1*-MO2 targeting the splice-donor site of exon6 (5′-AAATgtaagaacaagctccctt-3′, lowercase letters represent intronic sequence). Morpholinos and standard control oligonucleotide (MO-*control*) were injected into one-cell stage zebrafish embryos as previously described (Meder et al., 2011). Embryos were derived from mating transgenic (Tg) zebrafish containing a fluorescent EGFP reporter cassette driven by the heart-specific *myl7* promoter (*myl7:*EGFP Tg) and endothelial-cell-specific promotor *fli* (*fli:*dsRed Tg). MO-targeted (morphant) zebrafish were evaluated for defects in heart and muscular development compared to control-injected embryos from the same embryo clutch at 48 hpf, using fluorescence microscopy for visualization of EGFP and dsRed1. To confirm that the injected morpholinos specifically induced the defective phenotypes of morphants, we analyzed the MO-targeting region by PCR and a subsequent sequencing step using the morphant and control cDNA as template. Morpholino-modified antisense oligonucleotides used to generate specific splicing isoforms are listed in supplementary material Table S2.

### Functional assessment

Functional assessment of cardiac contractility was carried out as described before (Rottbauer et al., 2005). Fractional shortening of heart chambers was calculated with help of the zebraFS software (www.benegfx.de). Results are expressed as mean±s.d. Analyses were performed using an unpaired Student's *t*-test. A value of *P*<0.05 was accepted as statistically significant.

### RNA isolation and quantitative real-time PCR

Total RNA was isolated from 48 hpf control-injected and MO-injected zebrafish embryos and hearts using Trizol and chloroform and treated with DNaseI (Invitrogen, Carlsbad, CA) to digest contaminating genomic DNA. 60 ng of total RNA was reverse transcribed using a mixture of oligonucleotide (dT) and dNTPs and the SuperScript III first strand cDNA synthesis kit (Invitrogen, Carlsbad, CA). qRT-PCR was carried out according to standard protocols with the SYBR-Green method (Thermo Scientific, Waltham, MA) using an ABI 7000 system (ABI). Primers were designed using NCBI primer blast and synthesized by Metabion (Martinsried, Germany). The optimum annealing temperature for each primer set was determined prior to the analysis of experimental samples. The specificity of each primer set was monitored by dissociation curve analysis to ensure only a single product was amplified and that no primer dimers were formed. Supplementary material Table S3 details the genes validated by qRT-PCR and assay conditions. Threshold cycle (CT) values were recorded in the linear phase of amplification and the data were analyzed using the ΔCT method. *elfa* was used as the internal reference to further normalize the data. Results are presented as relative expression compared to control-MO-injected embryos.

### cDNA library preparation next generation sequencing

For library preparation, 4 to 6 µg of total RNA of the embryonic zebrafish heart samples and 9 to 25 µg of the zebrafish samples were enriched with the Ambion^®^ MicroPoly (A)Purist™ Kit to obtain all mRNA transcripts (refer to supplementary material Table S4). 500 ng of each sample were used for the fragmentation with RNase III. Purification of the fragmented RNA was performed by the RiboMinus™ Concentration Module (Invitrogen, Carlsbad, CA). SOLiD adaptors were ligated by adding the amounts listed in supplementary material Table S4 to the enriched fraction. After ligation, mRNAs were transcribed into cDNA with Array Script™ Reverse Transcriptase. cDNA fragments between 150 and 250 nt (mRNAs plus adaptors) were isolated from a 6% TBE Urea Gel (Novex-System, Invitrogen, Carlsbad, CA). RNA from gel slices was amplified with 16 PCR cycles using the same 5′-primer for each sample and six different 3′-primers including the barcode sequences (SOLiD Multiplexing Barcoding Kit 01-16). A total of six purified and barcoded DNA libraries were analyzed with a HS-DNA Chip in the Agilent Bioanalyzer 2100 and subsequently pooled in equimolar amounts.

### Next generation sequencing

The pooled libraries were diluted to a concentration of 60 pg/µl. The DNA was amplified monoclonally on magnetic beads in an emulsion PCR. Emulsions were broken with butanol and the remaining oil was washed off the templated double-stranded beads. DNA on the bead surface was denatured to allow hybridization of the enrichment beads to the single-stranded DNA. Using a glycerol cushion, the null beads can be separated from the templated beads. After centrifugation, the enriched magnetic beads were in the supernatant. The enrichment-beads were separated from the magnetic beads by denaturation. The 3′-end was enzymatically modified for deposition on the sequencing slide. Seven hundred million beads were loaded onto a Full Slide and sequenced on a SOLiD 4 analyzer (Applied Biosystems, Carlsbad, CA).

### Mapping reads to zebrafish genome and annotated genes

The reads obtained from sequencing were mapped to the zebrafish genome (*Danio rerio* zv8) using the BioScope software (Life Technologies, Darmstadt, Germany). In addition to the genomic sequence, the reference taken for mapping contained ribosomal and transfer RNA sequences from zebrafish, as well as sequences of the SOLiD adaptors and homopolymer sequences (poly-A, poly-T) were also used in order to filter reads matching these sequences. For the identification of reads covering exon-exon (inter-exon) junctions, sequences were added to the reference representing all possible combinations of two exons from each gene based on the zebrafish annotation (zv8; UCSC Genome Browser).

### Determination and normalization of gene expression levels

Expression levels of exons were determined according to their coverage by the sequencing reads. To enable cross sample comparison, coverages were normalized in terms of reads per kilobase of exon model per million mapped reads (RPKM) (Mortazavi et al., 2008).

### Determination of alternative splicing using RNA-seq

Splicing events were determined by the analysis of reads covering exon-exon junctions as identified by mapping to one of the two exon combinations added to the reference. The presence of splicing junctions in a sample was represented as the fraction of reads covering a particular exon-exon junction (parts per million; ppm) and related to the number of all reads found to map on exon junctions in that sample.

For the identification of splicing events removing single exons in only one of two samples (test or reference sample), we applied a function to each exon to calculate a score for its removal. The scoring was based on the coverages of exon-exon junctions (CoverageA, CoverageB, coverages of exon junctions in test or reference sample; i, exon number) as below:

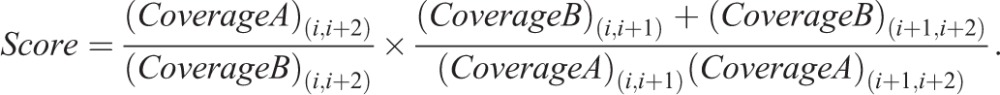



In order to identify splice-out events in both samples, the scoring function was applied twice after swapping the coverages for the two samples. Splice-out events were then selected for further examination based on the calculated score.

### Pathway analysis

To test whether genes with differential expression patterns were significantly accumulated in specific biochemical pathways, we applied the Gene Set Enrichment Analysis (GSEA) method for the differentially expressed genes in heart and whole-fish tissue based on the Kyoto Encyclopaedia of Genes and Genomes (KEGG) definition. GSEA produces an ordered list of genes as input and evaluates whether the genes on top of the list are enriched for participants in the respective pathways. To this end, we sorted all genes according to the absolute value of the median distances in heart and whole-fish tissue and uploaded the respective gene list to GeneTrail (Keller et al., 2008), a comprehensive web-based tool for various pathway analysis (http://genetrail.bioinf.uni-sb.de). GeneTrail incorporates all available KEGG pathways for *Danio rerio* and outputs significantly enriched pathways with respect to our input list.

## Supplementary Material

Supplementary Material
