# Supplementary material for: RNA splicing regulated by RBFOX1 is essential for cardiac function in zebrafish
Source: J Cell Sci. 2015 Aug 15;128(16):3030–40. doi: 10.1242/jcs.166850 (PMC4541041; doi:10.1242/jcs.166850)
Supplement: Supplementary Material [file supp_128_16_3030__index.html]

Supplementary Material 

# RNA splicing regulated by RBFOX1 is essential for cardiac function in zebrafish

## JCS166850 Supplementary Material

- Supplementary Material
