# Supplementary material for: RNA splicing regulated by RBFOX1 is essential for cardiac function in zebrafish
Source: J Cell Sci. 2015 Aug 15;128(16):3030–40. doi: 10.1242/jcs.166850 (PMC4541041; doi:10.1242/jcs.166850)
Supplement: Supplementary Material [file supp_jcs.166850_JCS166850supp.pdf]

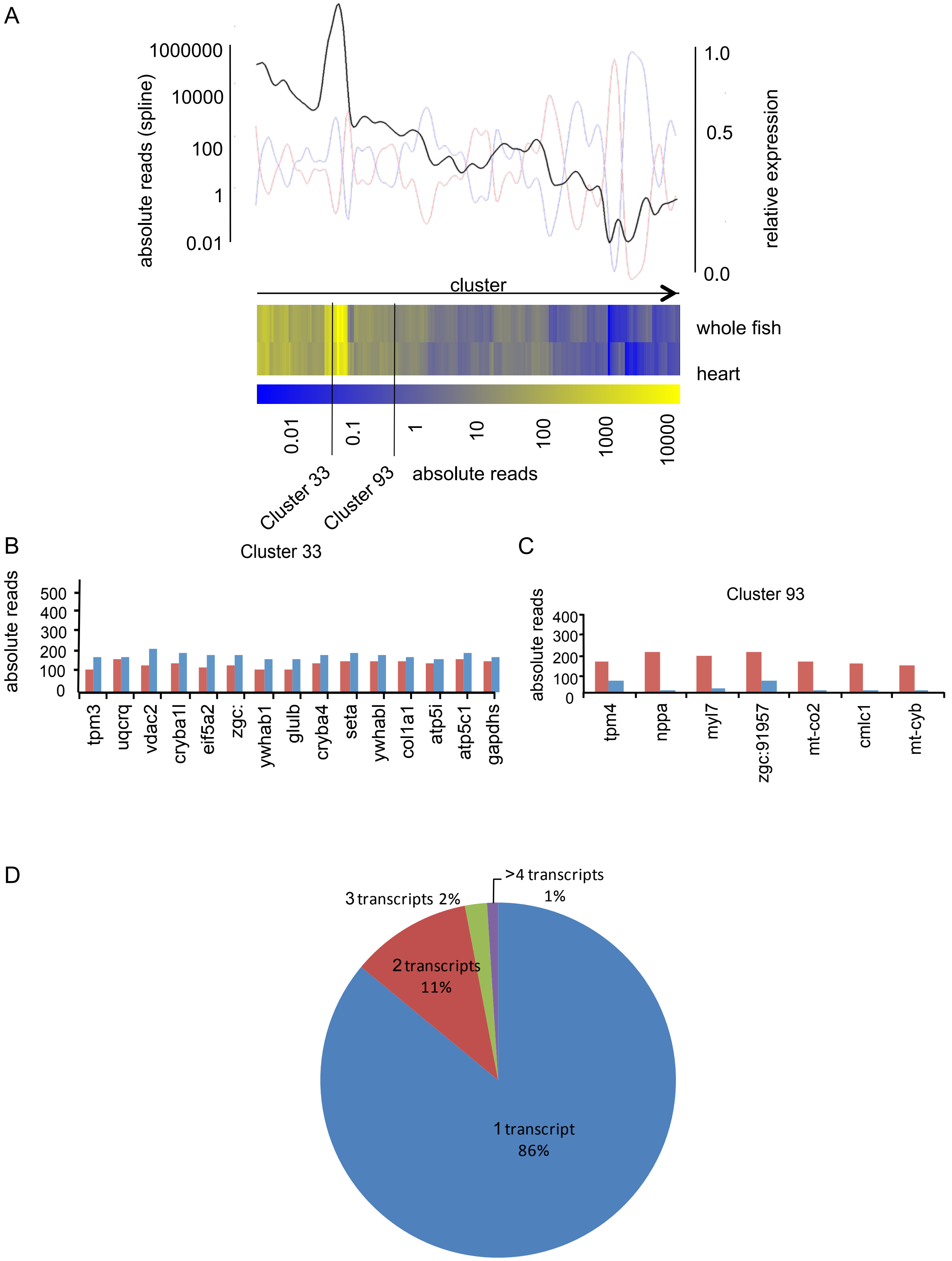
A
1000000
10000
100
1
0.01
absolute reads (spline)
1.0
0.5
0.0
relative expression
cluster
whole fish
heart
0.01
0.1
1
10
100
1000
10000
absolute reads
Cluster 33
Cluster 93
B
Cluster 33
absolute reads
500
400
300
200
100
0
tpm3
uqcrq
vdac2
cryba1l
eif5a2
zgc:
ywhab1
glulb
cryba4
seta
ywhabl
col1a1
atp5i
atp5c1
gapdhs
C
Cluster 93
absolute reads
400
300
200
100
0
tpm4
nppa
myl7
zgc:91957
mt-co2
cmlc1
mt-cyb
D
>4 transcripts 1%
3 transcripts 2%
2 transcripts 11%
1 transcript 86%

Fig. S1. Transcriptome profiling in zebrafish. (A) Schematic representation of the measured absolute reads and relative expression values of the identified transcripts in heart (red line) and whole embryo tissue (blue line). (B to C) Cluster of whole fish and cardiac specific gene expression patterns. Cluster 33 shows highly expressed genes in whole fish tissue whereas cluster 93 displays high expressed cardiac genes. Deep-RNA sequencing of zebrafish identified transcripts with one isoform (86%), with two (11%), with three (2%) and with four or more (1%) isoforms. No differences were observed between both tissues at this development stage

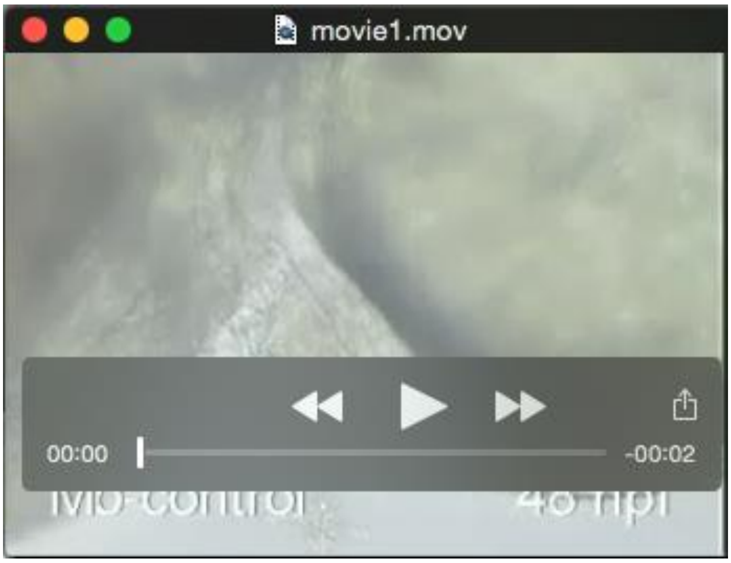

Movie 1. Control zebrafish embryos (48 hpf).
In MO-control-injected 48-hour old (72 hpf) zebrafish embryos both cardiac chambers contract vigorously. Lateral view, head to the left, ventral side downwards.

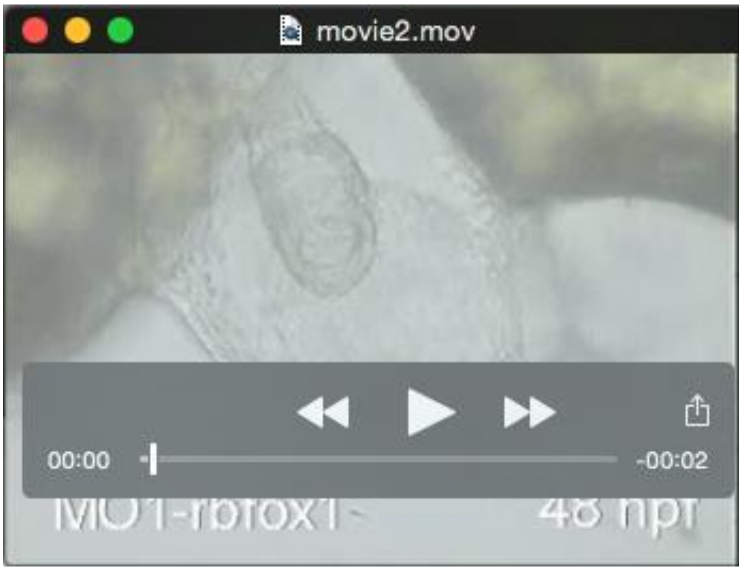

Movie 2. rbfox1-morphant zebrafish embryos (48 hpf).
In rbfox1-morphant embryos show significant atrial dilatation and a severely reduced contractility of both heart chambers. Lateral view, head to the left, ventral side downwards. Lateral view, head to the left, ventral side downwards.

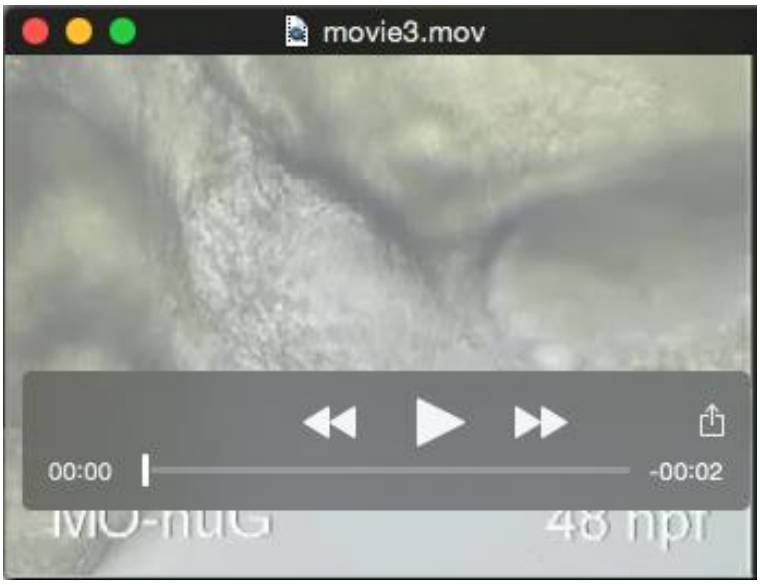

Movie 3. huG-morphant zebrafish embryos (48 hpf).
MO-*hug*-injected embryos develop heart failure with dilation of the both chambers and pericardial edema. In huG-morphant embryos cardiac contractile force is significantly reduced. Lateral view, head to the left, ventral side downwards.

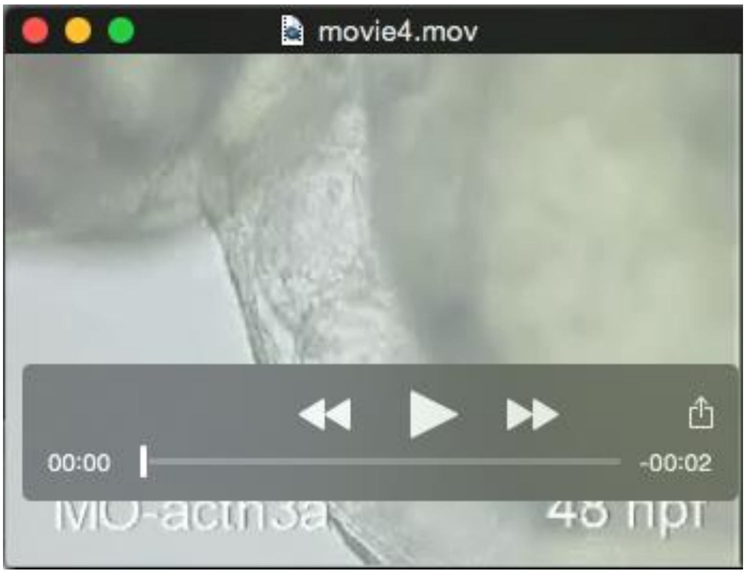

Movie 4. actn3a-morphant zebrafish embryos (48 hpf).
Actn3a-depleted zebrafish embryos develop heart failure with dilation of the atrium and reduced ventricular contractility. Additionally, *actn3a*-morphants show reduction of blood flow and pericardial blood congestion as consequence of the reduced cardiac function. Lateral view, head to the left, ventral side downwards

Table S1

Click here to Download Table S1

Table S2: Morpholino-modified antisense oligonucleotides

Gene	Gene ID	MO junction	MO sequence
actn3a	ENSDARG00000013755	I5/E6	TCCAACTAAGACACAGACCACAAAC
camk2g1	ENSDARG00000071395	E14/I14	GGTTACACAGAGATGAGGTACCATC
hug	ENSDARG00000016405	I2/E3	ACAAACCTGCAGCAACAGATCAGTT
ktn1	ENSDARG00000032802	I24/E25	AGCTCCTAGTCAGTTTCAGAGAAAC
ptpla	ENSDARG00000022633	E14/I14	GCTTCATGCCCCATTTGTACCTGGC

Table S3. Primer list.

Gene	Name	Sequence 5’- 3‘
a2bp1	a2bp1 ISP-F	CTGCAGGGATTGGGACTAAG
a2bp1	a2bp1 ISP-R	TTAATATGGCGCGAAACGAC
a2bp1	a2bp1 ISP-F2	ATGGAGGAAAAAGGGAGCAA
a2bp1	a2bp1 ISP-R2	TTAATATGGCGCGAAACGAC
a2bp1	a2bp1-splice fow	CATCGGCCCAGTTCGCTCCC
a2bp1	a2bp1-splice-rev	GATTTCAACATCTAAGATTTTACC
actn3a	actn3a-co-E2-fow	TGGTGCAACTCTCACCTGCGT
actn3a	actn3a-co-E2-rev	ACCTCCAGCAGCAGCATGAGC
actn3a	actn3a-E5	GACTGCCCCCTACAGGAACGT
actn3a	actn3a-E6 rev	ATGAGGGCACAAAGTGCCAGGC
actn3a	actn3a-E6 fow	AGACCTGACCTCATCGACTACTCCA
actn3a	actn3a-E7	GCAGTGTTGAGATTGCCAATTGGG
actn3a	actn3a-co-E2-fow	TGGTGCAACTCTCACCTGCGT
camk2g1	camk2g1-co-E6 fow	CCATTGCATCAGTCAGATCCTGGAG
camk2g1	camk2g1-co-E6 rev	AAGGTCTCTGTGCACGATGTCATGC
camk2g1	camk2g1 E13	CAAAAAGGCAGATGGAGTCAAG
camk2g1	camk2g1 E14 rev	TCGGTGTAGAAGAGGCCGTGTTG
camk2g1	camk2g1 E14 fow	CAACACGGCCTCTTCTACACCGA
camk2g1	camk2g1 E15	CTTTGGCCCATCGACGGGGTT
ktn1	ktn1-co-E4-fow	GGCTCATGTTCAGTCCTCTGCTCTG
ktn1	ktn1-co-E4-rev	TGGCAGGCGCTGGAACAGGA
ktn1	ktn1 E24	GGAAGAGCTGCTTGAGTTGAGGGA
ktn1	ktn1 E25 rev	GCGCCTCCATAGCGCTCCAG
ktn1	ktn1 E25 fow	CTGGAGCGCTATGGAGGCGC
ktn1	ktn1 E26	CGGTATGTGCGGCAGGAGTCTGT
hug	hug E5 co fow	GCCACAGTTTAGGGTACGGATTTGT
hug	hug E5 co rev	GGCCATTGAGTGTATTGATTGCCCT
hug	hug E2	TGTAAAGTGTCTACATGGCCGT
hug	hug E3 rev	GAGCTGGCACCCTTGTGAGAGT
hug	hug E3 fow	ACTCTCACAAGGGTGCCAGCTC

hug	hug E4	GCTCATCGCCCATGGGGTCC
ptpla	ptpla E1 co fow	CCTTCAGAAAGGCACCCATAAAGGC
ptpla	ptpla _E1 co fow	CTCCAGCAGTGCAAATGTCTGGA
ptpla	ptpla E3	TGGGGTCCAAGTGTGTTCTCGGA
ptpla	ptpla E5	CAGTTCACCAACTACTCCCAGTGGG

Table S4 : cDNA Library Preparation NGS

Sample	SID	Concentration	Working Concentration	Adapter Concentration
A2bp1 whole fish 48 hpf	BC13	393 ng/µl	25,4 µg	63 ng
Mock (a2pb1) whole fish 48 hpf	BC14	1100 ng/µl	9,1 µg	78 ng
A2pb1 heart 48 hpf	BC15	12,1 ng/µl	4,1 µg	145 ng
Mock heart 48 hpf	BC16	18,1 ng/µl	6,2 µg	48 ng

Table S5

Click here to Download Table S5
